# Imaging biomarkers and artificial intelligence for diagnosis, prediction, and therapy of macular fibrosis in age-related macular degeneration: Narrative review and future directions

**DOI:** 10.1007/s00417-025-06790-0

**Published:** 2025-03-10

**Authors:** Rishikesh Gandhewar, Thales Guimaraes, Sagnik Sen, Nikolas Pontikos, Ismail Moghul, Theodoros Empeslidis, Michel Michaelides, Konstantinos Balaskas

**Affiliations:** 1 Faculty of Medicine, Imperial College London, London, England; 2 Institute of Ophthalmology, University College London, London, England; 3 Moorfields Eye Hospital NHS Foundation Trust, 162 City Road, London, EC1V 2PD UK; 4 Stoneygate Eye Hospital, Leicester, England; 5 RAID Digital AI Reading Centre, Erie, PA USA

**Keywords:** Age-related Macular Degeneration, Macular Fibrosis, Anti-Vascular Endothelium Growth Factors, Biomarkers, Optical Coherence Tomography, Colour Fundus Photography, Fluorescein Angiography, Artificial Intelligence

## Abstract

Macular fibrosis is an end-stage complication of neovascular Age-related Macular Degeneration (nAMD) with a complex and multifactorial pathophysiology that can lead to significant visual impairment. Despite the success of anti-vascular endothelium growth factors (anti-VEGF) over the last decade that revolutionised the management and visual prognosis of nAMD, macular fibrosis develops in a significant proportion of patients and, along with macular atrophy (MA), is a main driver of long-term vision deterioration. There remains an unmet need to better understand macular fibrosis and develop anti-fibrotic therapies. The use of imaging biomarkers in combination with novel Artificial Intelligence (AI) algorithms holds significant potential for improving the accuracy of diagnosis, disease monitoring, and therapeutic discovery for macular fibrosis. In this review, we aim to provide a comprehensive overview of the current state of knowledge regarding the various imaging modalities and biomarkers for macular fibrosis alongside outlining potential avenues for AI applications. We discuss manifestations of macular fibrosis and its precursors with diagnostic and prognostic significance on various imaging modalities, including Optical Coherence Tomography (OCT), Colour Fundus Photography (CFP), Fluorescein Angiography (FA), OCT-Angiography (OCTA) and collate data from prospective and retrospective research on known biomarkers. The predominant role of OCT for biomarker identification is highlighted. The review coincides with a resurgence of intense research interest in academia and industry for therapeutic discovery and clinical testing of anti-fibrotic molecules.

## Key messages

*****What is known***:**

- Macular Fibrosis is a leading cause of medium- to long-term vision deterioration, following initial visual gains associated with the revolutionary effect of anti-vascular endothelial growth treatments for neovascular Age-related Macular Degeneration.
- Previously untreatable, a recent resurgence of intense academic interest, efforts in therapeutic discovery, and clinical trial pipelines for the evaluation of promising emerging treatments have generated renewed interest in the identification and validation of imaging biomarkers to serve as clinical trial endpoints.
- The advent of multimodal imaging and AI-enabled automated analytics has the potential for a paradigm shift in the efficiency for identification, quantification, and over-time monitoring of imaging biomarkers from complex imaging modalities, previously infeasible.

*****What is new***:**

- The review of various imaging modalities to inform the identification of the optimal imaging modality (or combinations of imaging modalities) for the application of automated analytics, and the efficient detection of suitable imaging biomarkers as clinical trials endpoints in Macular Fibrosis, is an essential first step for informing the role of imaging and AI-analytics in this potentially transformative field of research.
- Optical coherence tomography (alone or in combination with colour fundus imaging) has emerged as the imaging modality of choice for biomarker identification in macular fibrosis.
- In this narrative review of published literature, we are consolidating available evidence on the role of various imaging modalities for biomarker detection in macular fibrosis. Additionally, we are highlighting, through evidence and case examples, the prospect of AI-enabled technologies to accelerate biomarker identification across all stages of AMD, inform clinical trial design, and shorten the translational pipeline from therapeutic discovery to licensed vision-saving treatments.

## Introduction

Age-related macular degeneration (AMD) is a leading cause of visual impairment worldwide, predicted to affect 300 million people by 2040 and overrepresented in developed regions with ageing populations [1, 2]. Anti-vascular endothelial growth factor (VEGF) agents have revolutionised the treatment outcomes of neovascular AMD (nAMD) with regular intravitreal injections now forming conventional therapy. However, roughly 20% of patients will continue to suffer further losses in visual acuity (VA) and between 62–75% do not experience significant improvement of 3 + lines in clinical trials, with real-world outcomes often worse [3–7].

Macular fibrosis is an end-stage manifestation of nAMD and is the most significant negative prognostic factor for visual outcome, estimated to cause a quarter of patients to be treatment-resistant and leading to an average two lines of VA loss over 5 years [8–14]. Fibrosis is the result of a chronic inflammatory environment related to choroidal neovascularisation into the subretinal space, causing permanent destruction of retinal integrity [11, 15–17]. The terms Macular Fibrosis and Macular Scar are used inconsistently in the literature, mostly as synonyms but occasionally to signify severity of the manifestation with Macular Scar indicating the more severe and extensive forms. Cumulative prevalence of fibrosis is reported as high as 32% in the first year of treatment, rising to 46% at 2 years, with an annual incidence of up to 25% over the first two years of treatment and cumulative prevalence increasing to 56% at 5 years and 71% at 10 years [9, 10, 16, 18].

End-stage AMD manifestations (macular fibrosis and macular atrophy) have hitherto not been amenable to treatment with associated lack of targeted licensed treatments. However, emerging treatments that target Geographic Atrophy (GA) through cell-based therapies and pharmacologically modulating the complement cascade have reached phase 2 and 3 clinical trials—with the first intravitreal therapy recently approved [19]. This success has renewed optimism and intensified current efforts in tackling macular fibrosis[15].

A pre-requisite for effective clinical trial design and real-world patient management is the evidence-based selection of imaging biomarkers for reliable and repeatable diagnosis and monitoring treatment response, as well as to inform prediction modelling and patient stratification. This includes risk assessment for development of macular fibrosis in treated patients, macular fibrosis progression rate, visual impact, and treatment response. Clinical factors including age and best-corrected VA (BCVA) have demonstrated significant associations, however a refined understanding of imaging biomarkers will underpin both therapy discovery and real-world clinical management.

In this review, we summarise evidence from the scientific literature on ophthalmic imaging biomarkers, highlighting the predominant role of OCT-derived biomarkers. More recent work on OCT-angiography (OCT-A) and experimental polarisation-sensitive OCT (PS-OCT) indicating potentially informative novel biomarkers is discussed, yet assessment of their utility will require further investigation. We finally summarise the promising contributions of artificial intelligence (AI)- that have offered greater insight into the diagnostic and predictive significance of imaging biomarkers in both nAMD and GA pointing to similar future directions of investigation for extracting further value from macular fibrosis imaging through AI analytics.

## Methods of literature review

**Fig. 1:**
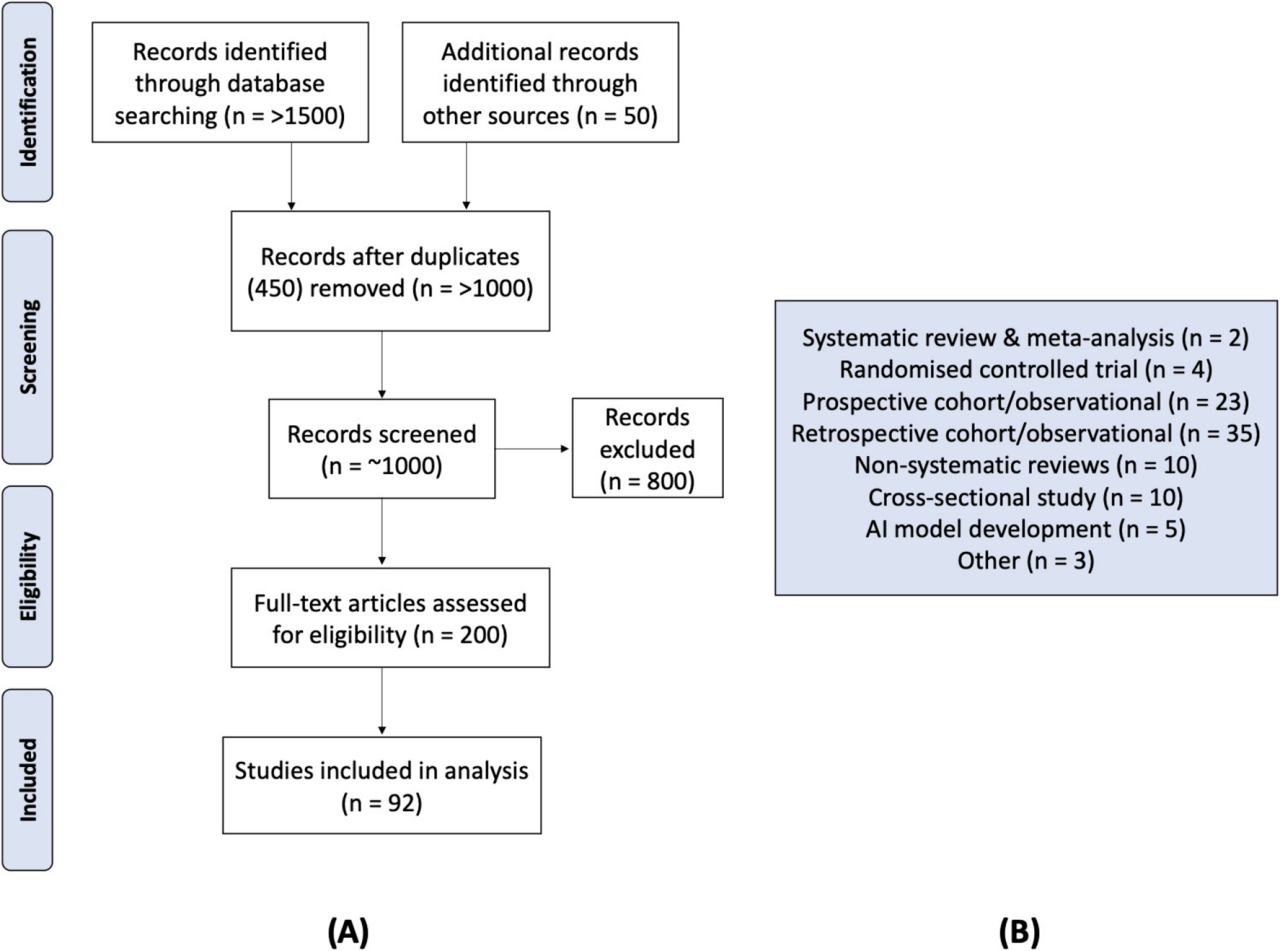
(**A**) Flowchart outlining literature review strategy (**B**) Summary of study types included

A comprehensive literature review was conducted to identify published articles related to clinical and imaging biomarkers for macular fibrosis. The MEDLINE Ovid (1946-present) and Embase Ovid (1947-present) databases were searched. We searched PubMed, Scopus, Science Direct, and IEEE Explore from inception to December 20, 2024, for studies in English that were likely to involve evidence on imaging biomarkers for macular fibrosis, as well as the role of AI in ophthalmic imaging for modelling in Age-related Macular Degeneration. Keywords within the search included: Neovascular (wet) age-related macular degeneration or macular fibrosis or macular scar in combination with biomarkers and imaging modalities, Optical Coherence Tomography (OCT), Fluorescein Angiography (FA), Colour Fundus Photography (CFP), (Subretinal) Hyper-Reflective Material, Intraretinal Fluid, Subretinal Fluid, Pigment Epithelial Detachment, Outer Retinal Tubulation and Artificial Intelligence. In excess of 1500 articles returned, after which nearly 450 duplicates were removed. All peer-reviewed titles and abstracts were screened. Further articles were identified through cross-references and citations. In total 200 articles were read in full, of which 125 made limited reference to imaging or they partially repeated evidence found in more comprehensive manuscripts. 92 papers were included and analysed (Fig. 1).

## Results

### Clinical biomarkers

Clinical biomarkers are relevant to identify baseline risk factors and contextualise imaging-based biomarkers. A meta-analysis of non-ocular risk factors for AMD identified increasing age, cigarette smoking and family history as highly significant risk factors for advanced AMD and increased body mass index and cardiovascular disease as significant [20].

Specifically for risk of fibrosis; age, gender and number of intravitreal injections had no significant influence on macular fibrosis development [21]. Furthermore, relevant genetic or serological biomarkers for fibrosis are yet to be established [22]. In 5-year follow up of participants of the CATT trial, baseline characteristics that were predictive of atrophic and fibrotic scar development included classic choroidal neovascularisation (CNV), good baseline VA in the other eye and the presence of large haemorrhage [18]. Similarly, other studies also identified classic CNV, poorer BCVA, longer interval before treatment initiation as predictive of vision loss of an additional 2 lines by 24 months [14, 21]. Overall, the value of clinical biomarkers in predicting progression to fibrosis is currently limited. Further prospective studies are required, in which the role of multi-modal AI methodologies could be effective for maximising the predictive value of clinical biomarkers through incorporating them in ensemble predictive modelling alongside imaging biomarkers.

### Ophthalmic imaging biomarkers

Various imaging modalities are used to diagnose, quantify, and monitor nAMD, including development of its sequelae such as macular fibrosis. These include colour CFP, FA, OCT, and OCTA. Historically, CFP has been the gold standard for macular fibrosis assessment, yet is inherently subjective, and even modern imaging with OCT and OCTA does not always allow distinct delineation of fibrosis [23]. AI may therefore prove significant in extracting additional value, especially from modern imaging modalities. Given its three-dimensional depth-resolved scanning and ubiquity in hospital eye departments globally, OCT provides greater granularity and insights into the imaging biomarkers of macular fibrosis as well as their diagnostic and prognostic significance, emerging as the modality of choice for the evaluation of macular fibrosis [24–29].

#### Colour fundus photography

CFP is the original imaging standard for monitoring retinal disease, including AMD. Its prevalence, ease of use and correlation with clinical examination mean it is still utilised in modern studies and provides a comparison to trials pre-dating modern imaging [30]. Macular fibrosis appears as a well-defined, elevated mound of yellow-whitish tissue that can be found in variable regions of the macula, yet fibrosis severity classification on CFP is highly subjective with poor inter-observer repeatability [31, 32]. macular fibrosis on CFP has been associated with larger baseline area of CNV and/or contiguous CNV- associated haemorrhage [18]. However, CFP underestimates the incidence of late AMD due to poor sensitivity; this is particularly true for early features of fibrosis, which are crucial for timely therapeutic intervention [32]. An intriguing correlation between black pigment and macular fibrosis has recently been described histologically, reviving interest in the role of CFP for fibrosis diagnosis and monitoring [33]. Black pigment is produced by melanotic cells, molecularly distinct from RPE cells, through transdifferentiation and occur more frequently in areas of pronounced macular fibrosis. Black pigment as a biomarker for macular fibrosis will require further investigation through multimodal imaging.

#### Fundus fluorescein angiography

Fundus fluorescein angiography (FFA) is a dynamic imaging technique that identifies vascular leakage and neovascularisation patterns. FFA has a minimal role in the diagnosis and monitoring of macular fibrosis, being most useful in the detection of type II (classical) CNV with a breached Bruch’s membrane, which is a known risk factor for fibrosis development. However, with the shift from laser and photodynamic therapy to anti-VEGF therapy, OCT has superseded FFA for serial monitoring [34, 35].

#### Fundus autofluorescence

Fundus Autofluorescence (FAF) has not been shown to contribute meaningful biomarkers in macular fibrosis. Fibrotic areas appear hypo-autofluorescent, often with a hyper-autofluorescent ring corresponding to an irregular, thickened RPE layer [36].

#### Optical coherence tomography

OCT has contributed immensely to the field of medical retina with its ability for non-invasive cross-sectional imaging at a near histopathological resolution [37]. nAMD is characterised by various OCT features including exudative fluid located at different retinal layers, pigment epithelial detachment (PED), tubulations and subretinal hyperreflective material (SHRM). These features have refined the understanding of nAMD and in prospective and retrospective analyses these biomarkers have been significantly associated with clinical outcomes, including BCVA, at both baseline and 12 months of treatment [14, 16, 18, 21, 38, 39].

##### Subretinal hyperreflective material

**Fig. 2:**
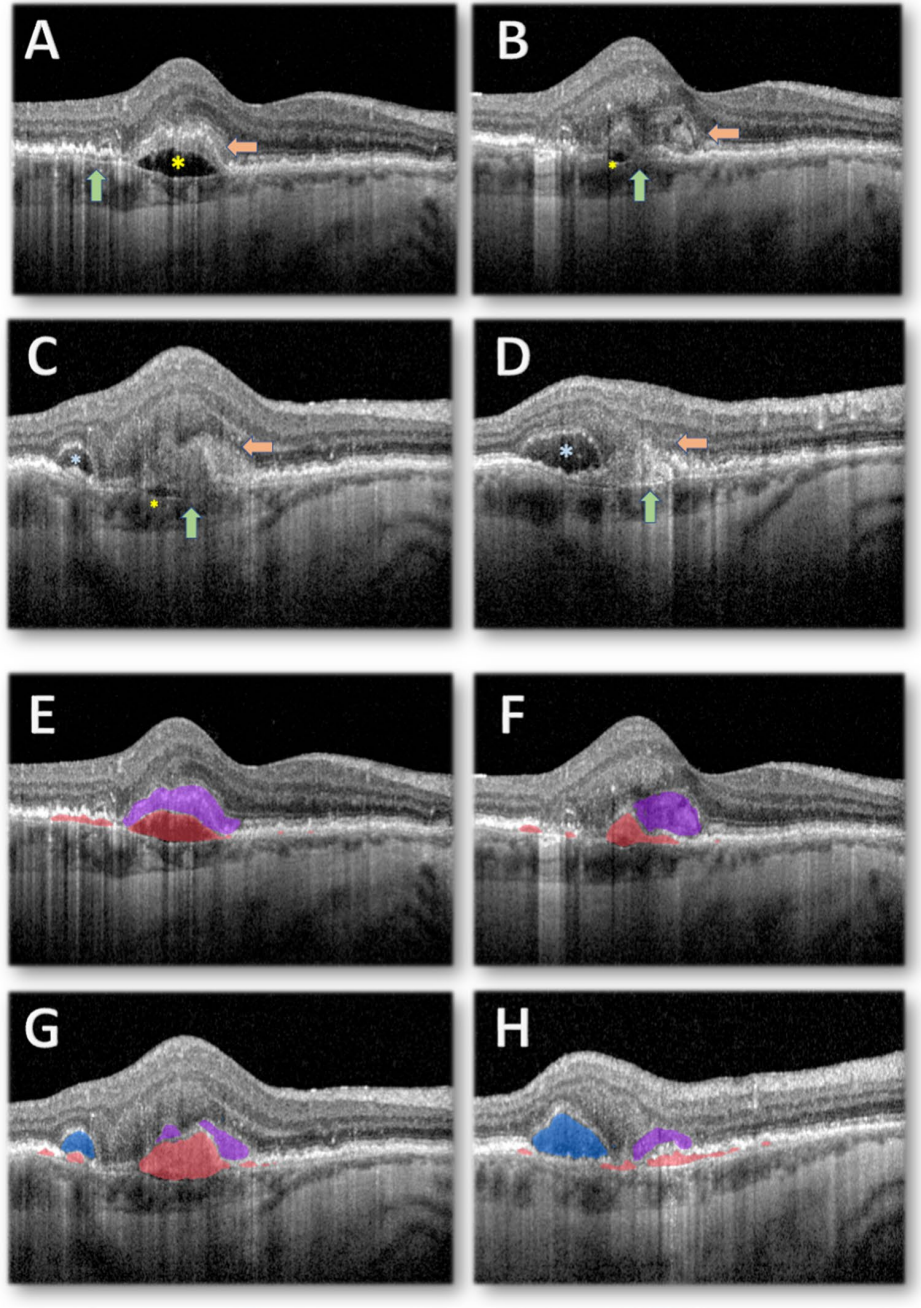
Optical Coherence Tomography b-scans from Baseline visit (pre-treatment) of patient with neovascular Age-related Macular Degeneration (nAMD) at Erie, PA (A-D). (**A**) b-scan 11, shallow PED with presence of SHRM and Sub-RPE HRM overlying Sub-RPE fluid. (**B**) b-scan 13, PED with HRM overlying a pocket of sub-RPE fluid with ill-defined SHRM. (**C**) b-scan 16, higher PED area with HRM overlying a small pocked of sub-RPE fluid and area of more clearly defined SHRM and presence of SRF. (**D**) b-scan 19, shallower area of hyper-reflective PED with higher reflectivity SHRM and modest definition with presence of SRF. Automated AI delineation of nAMD features* on corresponding OCT b-scans (E–H). (**E**) Shallower PEDs, SHRM and hyper-reflective PED, Sub-RPE fluid (**F**) PED with small pocket of sub-RPE fluid, larger area of SHRM (**G**) PED with hyper reflective content with small pocket of sub-RPE fluid, SHRM and SRF. Key. (A-D) Green arrow: Pigment Epithelium Detachments (PED), Orange arrow: Sub-Retinal Hyper-reflective Material (SHRM), Yellow asterisk: Sub-RPE Fluid, Blue asterisk: Subretinal fluid (SRF). (E–H) Pink area: PED, Purple area: HRM (including SHRM), Red area: Sub-RPE fluid, Blue area: SRF

**Fig. 3:**
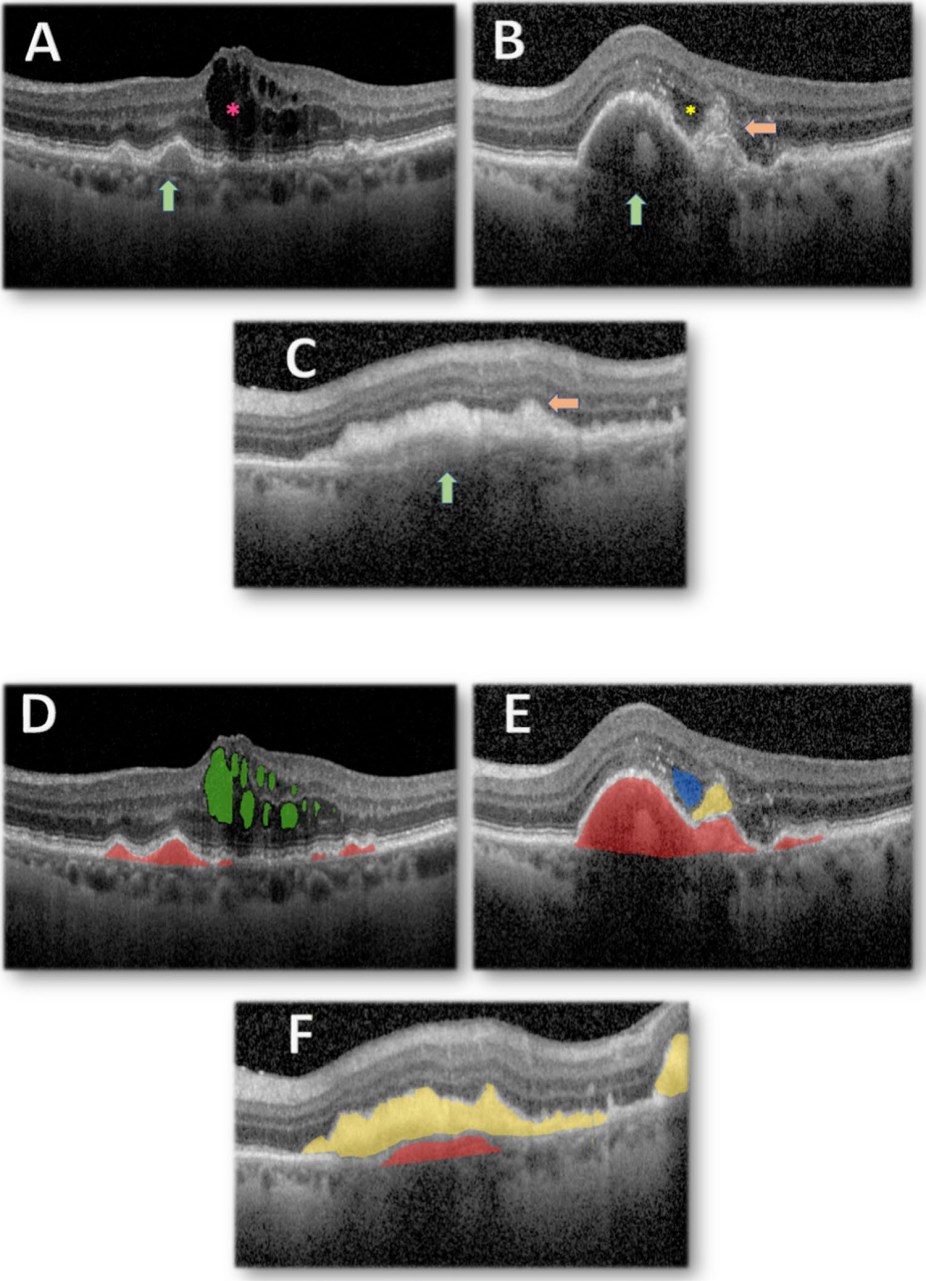
Central macular Optical Coherence Tomography b-scan from a patient with neovascular Age-related Macular Degeneration receiving treatment at Erie, PA (A-C). (**A**) Baseline visit, small fibrovascular PEDs with marked Intraretinal Fluid (IRF). (**B**) After 12 injections of anti-VEGF, large fibrovascular PED with area of marked hyper-reflectivity, SHRM and pocket of SRF. (**C**) After 36 injections of anti-VEGF, shallow PED with fibrotic content, extensive well-defined SHRM with increased reflectivity indicative of Macular Fibrosis. Automated AI delineation of nAMD features* on corresponding OCT b-scans (D-F). (**D**) IRF and PEDs (**E**) PEDs, SHRM and SRF (**F**) Shallow PED and extensive SHRM. Key. (A-D) Green arrow: Pigment Epithelium Detachment (PED), Orange arrow: Sub-Retinal Hyper-Reflective Material (SHRM), Pink asterisk: Intraretinal fluid (IRF), Yellow asterisk: Subretinal Fluid (SRF). (E–H) Green area: IRF, Red area: PED, Yellow area: SHRM, Blue area: SRF. *Automated OCT segmentation of nAMD features performed using proprietary deep-learning segmentation algorithm developed by the RAID Ophthalmic reading Centre, NV, USA. The report on the development and validation of RAID AI is under peer-review. Figures 2 & 3 are provided as illustrative examples of AI potential in the study of macular fibrosis

SHRM is an OCT-specific feature defined as areas of increased reflectivity, of variable shape, size, pixel intensity and sharpness of outline, located between the outer retina and RPE (Fig. 2 B-D, Fig. 3 B&C) [24–26]. SHRM is distinct from intraretinal lipids, exudates and hemorrhages, such as observed in type III CNV, where there is absence of a sub-RPE neovascular complex but a retino-retinal anastomosis in the early stages progressing to a retinochoroidal anastomosis, and SHRM may be the only sign of early nAMD [27, 28]. The precise contents of SHRM remain unclear, however it is suggested likely to be a combination of type II CNV elements, mixed CNV elements that have breached the RPE, red blood cells, fibrin, vitelliform material and fibrotic tissue [24, 25, 29].

In post-hoc analysis of the CATT trial, SHRM was found to be predictive of progression to fibrosis [16, 24]. Presence of SHRM on OCT was associated with FFA leakage and active CNV and can be identified prior to overt neovascularisation, as well as indicate CNV recurrence [26, 28, 40–42]. Owing to its association with fibrosis, SHRM may be one of the best correlated biomarkers for nAMD and long-term VA, with larger lesions predicting a worse outcome [21, 43–45]. Moreover, reduction in SHRM thickness and volume with anti-VEGF has been correlated with treatment efficacy; and its persistence could prove useful in predicting risk of fibrosis [11, 46]. Maximum SHRM width was the most significant prognostic factor for VA, although height and other morphology including reflectivity and SHRM definition have demonstrated associations and could signify an increasing fibrotic content compared to exudative component [28, 43, 46, 47]. Well-defined borders of SHRM predict a worse prognosis, along with a layered appearance and the presence of hyperreflective spots, which may be a feature of active inflammation [41, 45, 48]. However, a recent study has correlated hyperreflective material boundary remodelling (HRM-BR), or appearance of a well-defined hyperreflective inner boundary that separates persistent HRM from the neurosensory retina, to the functional outcomes associated with treatment with anti-VEGF, and it was noted that eyes with complete HRM-BR have better visual outcomes than eyes with partial or absent [49].

SHRM pre- vs post-treatment may be separate entities altogether, which may have significant prognostic importance [45]. Macular atrophy and fibrosis are different end points of nAMD, and they both have been shown to demonstrate SHRM on OCT, with the main difference being in thickness [50].

Hyperreflective material (HRM) has also been described in other locations. Intraretinal HRM is associated with type III CNV (Retinal Angiomatous Proliferation, RAP), whilst sub-RPE HRM constitutes a frequent component of type I CNV (sub-RPE neovascularisation with intact Bruch’s) and is part of the natural history of PED lesions, where it can be indicative of fibrotic content [47, 51]. In recent work, the predictive role of SHRM for the development of macular fibrosis was shown to be stronger for type III CNV [52].

Treated type I CNV complexes may regress to form residual sub-RPE HRM, leading to ring-shaped accumulation of depolarising material demonstrated by PS-OCT [53]. An intact RPE layer is essential for this process and may protect against fibrosis; as conversely eyes with type II CNV associated subretinal fibrosis demonstrate a discontinuous RPE layer in fibrotic regions [12, 21]. Similarly, increased space between SHRM and neuroretina was a positive prognostic indicator, with the subretinal fluid (SRF) possibly acting as a protective layer [45, 48, 54].

Overall, HRM, and particularly SHRM, could be a pivotal biomarker for the prediction and monitoring of fibrosis [55]. Further prospective research is required to corroborate current findings and develop objective outcome measures. Modern image analytics through deep-learning methodologies may help to quantify these trends and unpick the significance of its dynamic and nuanced morphology.

##### Intraretinal fluid and subretinal fluid

Retinal fluid is caused by exudation and is therefore a criterion for CNV and nAMD diagnosis. It is initially recognised clinically and can be classified as SRF and intraretinal fluid (IRF), which (location-dependent) may contribute towards central retinal thickness (CRT). In the CATT trial, 82% of patients had fluid involving the fovea and 30% had fluid in all compartments (intra-, sub-retinal and sub-RPE) [56].

IRF is largely considered as the most important negative risk factor for worsening vision in nAMD and is associated with type II and mixed CNV [44, 57]. At baseline it correlates strongly with VA and poorer treatment response, with volume and location having further influence [58]. Reducing IRF with therapy generally demonstrated a linear improvement in vision [58]. Patients with IRF at any treatment stage are at greater risk of fibrosis, including at baseline and in cases of persistent IRF despite a year of injections [21, 44, 57, 59]. (Fig. 3, A-C) Similarly, fluctuations in retinal fluid (and particularly IRF) are associated with significantly worse VA after 2 years [60, 61].

Some studies have shown SRF to be a positive prognostic marker in treated eyes [44], with its absence showing lower progression to fibrosis. However, this is not universally accepted and may relate to prompt initiation with anti-VEGF therapy, which modulates disease behaviour [21, 59]. The significance of sub-RPE fluid for the development of fibrotic PEDs or macular fibrosis remains unclear (Fig. 2, A-C).

AI-enabled segmentation is already employed for detailed volumetric quantification of IRF and SRF (as well as PED and SHRM). Using prediction models, their association with fibrosis may be better understood [60] (Fig. 2, E–H).

##### Outer retinal tubulation

Outer retinal tubulation (ORT) refers to a branching tubular structure found in the outer nuclear layer, thought to represent a retinal response to progressive photoreceptor damage [62–64]. Its reported prevalence ranges from 17.4% to 41.6% in studies with up to 4-years follow up [16, 18, 65, 66]. The CATT study found a correlation between ORT and worsening VA, but other studies have reported the opposite, with ORT being associated with stability [65, 67]. ORT in various other studies was found to be associated with SHRM, subretinal fibrosis and loss of sub-foveal photoreceptor integrity, ultimately leading to worse VA [23, 68].

##### Pigment epithelial detachment & retinal pigment epithelial tears

PED is defined as separation of the RPE from the inner layer of Bruch’s membrane (Fig. 3, A&B) and can be present in up to 80% of nAMD eyes [44]. Despite its high prevalence there is an inconsistent reported association with visual outcomes [69]. PED is thus considered less relevant than retinal fluid in predicting response, and rather its value may lie in combination with other biomarkers [44].

The natural history of PED is occasional development of an RPE tear in roughly 10% of eyes [70, 71]. Small lesions have a propensity to self-heal, whilst large lesions may lead to fibrosis [44, 72]. Spontaneous RPE tears can also be associated with massive fibrovascular scarring, noted in roughly 30% of long-term follow up cases [70, 72].

The association between Sub-RPE HRM and macular fibrosis has been discussed in the SHRM subsection. In advanced stages, the concurrent development of fibrotic SHRM (representing macular fibrosis) and fibrotic PED often renders the boundary between the subretinal and sub-RPE spaces indistinguishable. In these common cases, a consensus is lacking on whether the entire hyper-reflective fibrotic lesion should be considered as macular fibrosis (Fig. 3C).

##### Outer retinal changes

Other potentially valuable biomarkers in prediction of VA and fibrosis include hyperreflective splitting of the RPE, which eventually ‘envelops’ the regressing SHRM after treatment, and the observed distinct hyperreflective line above the SHRM that also seems to wane with treatment [22, 45, 73, 74]. Moreover, better functional outcome has been reported with intact ellipsoid zone-external limiting membrane complex [55]. Local predisposing factors for macular fibrosis development include a combination of overlapping increased RPE thickness plus drusen plus neovascular lesions complex, in some studies abbreviated as RPEDLC [39, 75]. However, it has also been reported that the majority of pixels from areas of macular fibrosis on ophthalmic imaging can be tracked back to pixels of a macular neovascular membrane at the same location, indicating an intimate pathophysiological correlation of fibrosis with the CNV complex. [39].

#### Optical coherence tomography angiography

OCTA is a non-invasive imaging technique that permits visualisation of retinal and choroidal vasculature, including blood flow signals and physiological versus pathological vessels [21, 76]. The size of a CNV lesion detected on OCTA is seen to be larger in eyes with subretinal fibrosis at baseline [77]. OCTA may also prove helpful to study neovascular remodelling in eyes with fibrosis, especially for those with suboptimal treatment response. OCTA studies have documented presence of blood flow in 94% of macular fibrosis, with OCTA uniquely enabling residual neovascular membrane components with active blood flow to be distinguished from normal choroidal vasculature [31]. Moreover, residual vascularity in macular scars has been associated with improved visual outcomes, as they may represent the only remaining source of blood supply in an area of ischaemia [78].

Subretinal fibrosis can be seen on OCTA as dense hyperreflective thickened bands and can provide a background contrast against which residual CNV components become more easily discernible [78, 79].

The application of OCTA in imaging and clinical studies will likely further our understanding of fibrosis pathophysiology and specifically vascularity in fibrosis.

#### Novel imaging modalities

PS-OCT is a modality that provides additional contrast to analyse the polarisation state of light, which is influenced by birefringent or depolarising material [21, 80]. Fibrotic material appears birefringent allowing for further opportunity to monitor its presence with specific capability to distinguish from the RPE and further discriminate SHRM into component elements [80–83]. Early models have confirmed proof-of-concept, however optimisation is required for further use [81, 84, 85].

### Role of artificial intelligence for imaging biomarkers’ identification

There is a growing role of AI systems, in particular deep-learning models, for automated segmentation of ophthalmic imaging, including entire volume OCT scans. These have significant capability for furthering our understanding of disease progression and for creating diagnostic and predictive models. Deep-learning automated segmentation allows quantification of imaging biomarkers, including three-dimensional volumetric measurement of features, as well as their evolution longitudinally, topographically and in response to treatment (Fig. 3, D-F) [86, 87]. This has enabled additional insight that was previously only possible through labour-intensive and time-consuming manual segmentation of small datasets by expert graders in specialist reading centres.

The potential of AI has already been demonstrated in various forms of AMD, including nAMD and GA. Zhang et al. developed and externally validated a deep-learning model for the detection, quantification, monitoring, and classification of macular atrophy following the Consensus Definitions for Atrophy (CAM) group classification [88]. The model performed at the level of human expert graders volume manual OCT segmentation) [88]. Deep-learning models have also been developed for nAMD biomarkers including IRF, SRF, PED and SHRM (Fig. 2 E–H, Fig. 3 D-F). These have demonstrated applicability in diagnosing and triaging retinal disease as well as predicting conversion to nAMD, which exceeded inter-observer variability in human expert predictions [87–91].

Machine learning applications in GA have been applied to successfully predict BCVA, demonstrating the potential for AI-generated predictive biomarkers [92]. Such models assess the relative importance of multiple imaging biomarkers in clinical outcomes and elucidate further structure–function correlations.

Rigorously evaluated, generalisable and high-performing AI models can facilitate not only the development and validation of imaging biomarkers in AMD, but also the standardisation of their interpretation and quantification, rendering the results of clinical trials on emerging therapeutics directly comparable. For instance, the CATT and HARBOR trials used OCT findings as re-treatment criteria, requiring clinical interpretation. In post-hoc analysis, disagreement in fluid identification between clinicians and reading centre was as high as 27.9% [93].

When considering macular fibrosis, machine learning based approaches for predicting fibrosis development have been recently described [94]. Efforts to-date have not used convolutional neural networks (deep learning) and have employed more conventional predictive modelling (random forests, xgBoost). Such efforts are limited in that they are not informed by automated segmentation and quantification of pathological disease features on ophthalmic imaging (imaging biomarkers), as is the case for other aspects of AMD (neovascular AMD, GA). Further studies including deep learning and automated OCT segmentation will be needed for quantification and morphological phenotyping of SHRM. Such an analytical approach would potentially facilitate the identification of suitable diagnostic and prognostic biomarkers, enabling efficient clinical trial design for emerging therapeutics. Future trials could potentially benefit from AI-enabled voxel-level segmentation to develop more sophisticated and clinically relevant biomarkers, as demonstrated in GA models. Potential models could rapidly quantify volume of critical features, including SHRM and IRF, and monitor them longitudinally. This is an area of active research interest of our research group. We are providing figures of automated segmentation outputs from research models currently undergoing validation and peer-review. Here, these are provided for illustrative purposes only. More automated analysis of large-scale data has become feasible across entire trial datasets and this will help establish key endpoints and predictive trends for future clinical trials.

## Conclusions

Neovascular AMD continues to represent a significant global cause of preventable vision loss, despite the effective control of active exudation. Whilst anti-VEGF has greatly improved visual outcomes, a significant proportion of patients still progresses to fibrosis and irreversible vision loss. Currently, there are no available agents to limit or reverse fibrosis, however the search for novel therapeutics is intensifying.

To further our understanding of fibrosis, manage and predict its course, and maximise value of clinical trial data, it is essential that objective imaging biomarkers are established. Traditional imaging modalities have largely been superseded by advancements in OCT, which has identified several promising biomarkers for macular fibrosis. SHRM has the strongest correlation with fibrosis to date, whereas other features including retinal fluid, ORT, PED and RPE tears have a less concrete association and require further exploration. The position of OCT as the imaging modality of choice for investigating macular fibrosis is becoming increasingly established. In a recent comparative study of OCT, CFP and FFA in macular fibrosis, the authors conclude that “Well-defined HRM detected by SD-OCT during the maintenance phase of anti-VEGF therapy explains BCVA variance, positioning OCT as a superior standard for detecting macular fibrosis”, suggesting a potential structure–function correlation. [95].

Artificial Intelligence has enabled the development of efficient and reliable systems for the automated analysis of ophthalmic imaging, and particularly OCT, with much improved time- and cost-efficiency. Such systems have been developed, validated and licensed for use in clinical settings supporting diagnosis, monitoring and prognosis of most AMD stages. The benefits of AI for identification of imaging biomarkers as optimal clinical trial endpoints are being leveraged in clinical trial designs for the treatment of end stage AMD manifestations, such as GA. The same techniques are being refined and used, including by our research group, to support intensifying research efforts in macular fibrosis.
